# Quantitative Changes in the Mitochondrial Proteome of Cerebellar Synaptosomes From Preclinical Cystatin B-Deficient Mice

**DOI:** 10.3389/fnmol.2020.570640

**Published:** 2020-11-13

**Authors:** Katarin Gorski, Albert Spoljaric, Tuula A. Nyman, Kai Kaila, Brendan J. Battersby, Anna-Elina Lehesjoki

**Affiliations:** ^1^Folkhälsan Research Center, Helsinki, Finland; ^2^Department of Medical and Clinical Genetics, Medicum, University of Helsinki, Helsinki, Finland; ^3^Molecular and Integrative Biosciences, and Neuroscience Center (HiLIFE), Faculty of Biological and Environmental Sciences, University of Helsinki, Helsinki, Finland; ^4^Institute of Clinical Medicine, University of Oslo and Oslo University Hospital, Oslo, Norway; ^5^Institute of Biotechnology, University of Helsinki, Helsinki, Finland

**Keywords:** myoclonus epilepsy, synaptosome, neurodegeneration, cerebella, electrophysiology, mitochondria

## Abstract

Progressive myoclonus epilepsy of Unverricht-Lundborg type (EPM1) is a neurodegenerative disorder caused by loss-of-function mutations in the cystatin B (*CSTB*) gene. Progression of the clinical symptoms in EPM1 patients, including stimulus-sensitive myoclonus, tonic-clonic seizures, and ataxia, are well described. However, the cellular dysfunction during the presymptomatic phase that precedes the disease onset is not understood. CSTB deficiency leads to alterations in GABAergic signaling, and causes early neuroinflammation followed by progressive neurodegeneration in brains of a mouse model, manifesting as progressive myoclonus and ataxia. Here, we report the first proteome atlas from cerebellar synaptosomes of presymptomatic *Cstb*-deficient mice, and propose that early mitochondrial dysfunction is important to the pathogenesis of altered synaptic function in EPM1. A decreased sodium- and chloride dependent GABA transporter 1 (GAT-1) abundance was noted in synaptosomes with CSTB deficiency, but no functional difference was seen between the two genotypes in electrophysiological experiments with pharmacological block of GAT-1. Collectively, our findings provide novel insights into the early onset and pathogenesis of CSTB deficiency, and reveal greater complexity to the molecular pathogenesis of EPM1.

## Introduction

Progressive myoclonus epilepsy, EPM1 (Unverricht-Lundborg disease; OMIM 254800) is an autosomal recessive neurodegenerative disorder characterized by disease onset between 6 and 16 years, severe stimulus-sensitive, treatment-resistant and physically disabling myoclonus, tonic-clonic seizures and ataxia, with cognition essentially preserved (Koskiniemi et al., 1974; Kälviäinen et al., 2008). Magnetic resonance imaging (MRI) studies of EPM1 patient brains have shown loss of gray matter volume in the thalamus and motor cortex (Koskenkorva et al., 2009), thinning of the sensorimotor, visual and auditory cortices (Koskenkorva et al., 2012), and widespread degenerative white matter changes (Manninen et al., 2013). The histopathology have revealed widespread non-specific degenerative changes in both the cerebellum and cerebrum (Haltia et al., 1969; Koskiniemi et al., 1974; Eldridge et al., 1983; Cohen et al., 2011). MRI-navigated transcranial magnetic stimulation studies have shown significant neurophysiological changes in cortical responses, including increased prevailed inhibition in the primary motor cortex (Danner et al., 2009) and impaired intracortical interactions and coherence (Julkunen et al., 2013).

EPM1 is caused by loss-of-function mutations in cystatin B (*CSTB*; OMIM 601145) (Pennacchio et al., 1996; Joensuu et al., 2008). The majority of patients are homozygous for a repeat expansion mutation in the promoter region of *CSTB*, reducing CSTB protein expression to 5–10% of controls (Joensuu et al., 2007). When patients are compound heterozygous for the repeat expansion and a null mutation, a more severe clinical presentation of EPM1 manifests (Koskenkorva et al., 2011; Canafoglia et al., 2012). Patients homozygous for predicted null mutations in *CSTB* manifest a severe neonatal-onset progressive encephalopathy (Mancini et al., 2016; O’Brien et al., 2017), suggesting an important role for CSTB in nervous system development. CSTB is a ubiquitously expressed intracellular inhibitor of cysteine proteases, including cathepsins B, H, S, K, and L (Turk et al., 2008). In addition, it is reported to protect cells against apoptosis (Pennacchio et al., 1998) and oxidative stress (Lehtinen et al., 2009), and play a role in cell cycle regulation (Ceru et al., 2010). Nonetheless, the precise molecular function of CSTB at the cellular level is far from resolved.

A genetically modified mouse model with a complete loss of *CSTB* expression (*Cstb*^–/–^) manifests myoclonus from one month and a progressive ataxia from 6 months of age (Pennacchio et al., 1998). The onset of the clinical symptoms are preceded by early glial activation and inflammation, followed by progressive volume loss in the brain and increased oxidative damage (Lehtinen et al., 2009; Tegelberg et al., 2012; Okuneva et al., 2015). Pathological changes are detected throughout the brain, but are most striking in the cerebellum (Pennacchio et al., 1998; Tegelberg et al., 2012), where progressive atrophy reduces the cerebellar volume by almost 50% at 6 months of age (Tegelberg et al., 2012).

The characteristic symptoms in EPM1 imply alterations in the inhibitory GABAergic circuits of the brain. In older *Cstb*^–/–^ mice, this presents as decreased GABAergic inhibition in the cortex (Buzzi et al., 2012), higher susceptibility to kainate-induced seizures (Franceschetti et al., 2007), and a progressive loss of inhibitory interneurons (Buzzi et al., 2012). However, altered GABAergic signaling was first noted in cerebella of presymptomatic postnatal day (P) 7 aged *Cstb*^–/–^ mice during GABAergic synapse development (Joensuu et al., 2014). This presented as increased expression of the genes encoding for GABA_A_ receptor (GABA_A_R) subunits α6 and δ in cerebellar tissue lysates, and as an imbalance between excitatory and inhibitory postsynaptic currents (EPSCs; IPSCs), and a complete absence of synchronous IPSC bursts in cerebellar Purkinje cells. At this early age, the number of interneurons in the cerebellum was not altered. By the early symptomatic stage (P30), the number of GABAergic presynaptic terminals and ligand binding to GABA_A_Rs were reduced. Together, the data indicate a disruption of GABAergic synapse formation in *Cstb*^–/–^ mice.

To investigate the mechanisms behind the altered GABAergic synapse formation, we used a quantitative mass-spectrometry-based proteomics approach to analyze cerebellar synaptosomes of presymptomatic *Cstb*^–/–^ mice. The data show a robust alteration to the mitochondrial proteome and factors important for intracellular transport and cytosolic ribosomal biogenesis.

## Materials and Methods

### Ethics Statement

The Animal Ethics Committee of the State Provincial Office of Southern Finland approved all animal research protocols (decisions ESAVI/10765/2015 and ESAVI/471/2019).

### Mice

*Cstb*^–/–^ mice were obtained from The Jackson Laboratory (Bar Harbor, ME; 129-Cstb^tm1Rm^/SvJ; stock #003486) (Pennacchio et al., 1998). Age-matched wild-type (wt) mice of the 129S2/SvHsd background were used as controls. The *Cstb*^–/–^ mouse line was maintained by backcrossing heterozygous *Cstb*^–/–^ males with inbred wt females and expanding the colony from heterozygous littermates.

### Proteomics

#### Synaptosome Isolation

Synaptosomes were isolated from the cerebella of P14-aged *Cstb*^–/–^ and wt mice (*n* = 5/genotype, males and females) according to the protocol described by Nolt et al. (2011), with slight modifications. Briefly, mice were sacrificed by decapitation and the cerebella dissected into ice-cold PBS. Cerebella were homogenized in 3 ml ice-cold buffer [0.32 M sucrose, 4 mM Hepes pH 7.4, protease- and phosphatase inhibitors (Pierce Protease and Phosphatase Inhibitor Mini Tablets, Thermo Fisher Scientific, Waltham, MA, United States)] and centrifuged twice at 1000 × *g* for 10 min at +8°C. The resulting supernatant was centrifuged at 10 000 × *g* for 15 min at +8°C. The resulting pellet was resuspended in 1.5 ml homogenization buffer and re-centrifuged to yield a washed crude synaptosomal fraction. This fraction was resuspended in 2 ml homogenization buffer and layered on top of 10 ml ice-cold 1.2 M sucrose and centrifuged at 230 000 × *g* (Sw40Ti swinging bucket rotor, Optima L-80 XP ultracentrifuge, Beckman Coulter, CA, United States) for 15 min at +4°C. The gradient interphase was diluted in homogenization buffer, layered on top of 9 ml ice-cold 0.8 M sucrose, and centrifuged at 230 000 × *g* for 15 min at +4°C. Synaptosomal purity and protein enrichment were analyzed by Western blot (Supplementary Figure S1).

#### Sample Preparation and LC-ESI-MS/MS Analysis

Lipids were removed from synaptosome samples by incubating them overnight at −20°C in five volumes ice-cold (−20°C) acetone. Samples were centrifuged twice at 1000 × *g* for 10 min at +8°C with an additional acetone wash in between. The pellet was air dried for 5 min and resuspended in freshly prepared 6.0 M urea/25 mM ammonium bicarbonate. Protein concentrations (μg/μl) were determined spectrophotometrically using the BCA protein assay kit (Pierce, Thermo Fisher Scientific) according to the manufacturer’s instructions. For analysis of the proteome, 25 μg of each sample was digested with trypsin (1:30 w/w, enzyme:protein; V5111 Sequencing Grade Modified Trypsin, Promega Corporation, WI, United States), and desalted by C18 cartridges. An additional phospholipid removal step was applied to samples due to impurities that interfered with ionization. Phospholipids were removed by hydrophilic interaction liquid chromatography (HILIC) using HILIC HyperSep Tips (60109-214; Thermo Fisher Scientific). Tips were conditioned by aspirating/expelling 50 μl of binding solution [15 mM ammonium acetate, pH 3.5 in 85% acetonitrile (ACN)] for five times prior to sample binding, and peptides were bound to the HILIC tips by aspirating/expelling the samples (10 μl, diluted in 85% ACN) for 20 times. Samples were washed by aspirating/expelling 20 μl binding solution for ten times, discarding the expelled solution each time. Peptide samples were released by aspirating/expelling 10 μl elution buffer (15 mM ammonium acetate, pH 3.5 in 10% ACN) for ten times, collecting the expelled solution in a 1.5 ml Eppendorf Maximum Recovery Tube (Axygen, Corning, NY, United States). Purified peptide samples were concentrated (SpeedVac, Thermo Fisher Scientific) for 10–15 min and dissolved in 1% formic acid. For liquid chromatography-electrospray ionization-tandem mass spectrometry (LC-ESI-MS/MS), 400 ng peptides/sample were analyzed in random order with several washes and blank runs in between. The analyses were performed on a nanoflow HPLC system (Easy-nLC1000, Thermo Fisher Scientific, Bremen, Germany) coupled to a Q Exactive mass spectrometer (Thermo Fisher Scientific) equipped with a nano-electrospray ionization source. Peptides were loaded on a trapping column and separated inline on a 15 cm C18 column (75 um × 15 cm, ReproSil-Pur 5 um 200 Å C18-AQ, Dr. Maisch HPLC GmbH, Ammerbuch-Entringen, Germany), with the mobile phase consisting of water with 0.1% formic acid (solvent A) and ACN/water [80:20 (v/v)] with 0.1% formic acid (solvent B). Peptides were eluted with a two-step linear gradient at a flow rate of 300 nl/min: from 2 to 20% of solvent B in 85 min, and to 40% of solvent B in 35 min, followed by a 15 min wash with 100% solvent B. MS analysis was performed using the Thermo Xcalibur 3.0 software (Thermo Fisher Scientific). The analyses were performed in a data-dependent acquisition (DDA) mode for 10 most intense peptide ions, consisting of an Orbitrap MS survey scan of mass range 300–2000 m/z, with a resolution of 140 000, mass window for precursor ion selection 2.0 m/z, and intensity threshold for triggering MS2 240, followed by fragmentation of selected peptide ions (MS2) by higher-energy collisional dissociation (HCD), with a mass resolution of 17 500 for MS/MS. Ions with unassigned charges and singly charged ions were excluded for precursor selection. The selected peptide ions were fragmented with a normalized collision energy of 27 in the collision cell. Dynamic exclusion duration was 10 s.

#### MS Quantification and Protein Identification

Peptides were quantified using the Progenesis LC-MS software (Non-linear Dynamics Limited, Tyne, United Kingdom), and proteins were identified using the Mascot 2.4.1 search engine (Matrix Science, MA, United States) through the Proteome Discoverer 1.4 software (Thermo Fisher Scientific). The Swiss-Prot Mus musculus database, uploaded in January 2016, was used as reference. Error tolerances on peptide and fragment ions were maximum 5 ppm and 0.02 Da, respectively. Database searches were limited to fully tryptic peptides with maximum one missed cleavage, and cysteine carbamidomethylation (+57.021464 Da) and methionine oxidation (+15.994915 Da) were set as fixed and variable modifications, respectively. Peptide-level false discovery rate (FDR) was set to 1%, and minimal peptide length to 7 amino acids. Only proteins with minimum two unique peptides were used for further analyses. The mass spectrometry proteomics data have been deposited to the ProteomeXchange Consortium^1^ via the PRIDE partner repository (Vizcaíno et al., 2013) with the dataset identifier PXD019370.

#### Data Processing and Analysis

**FIGURE 1:**
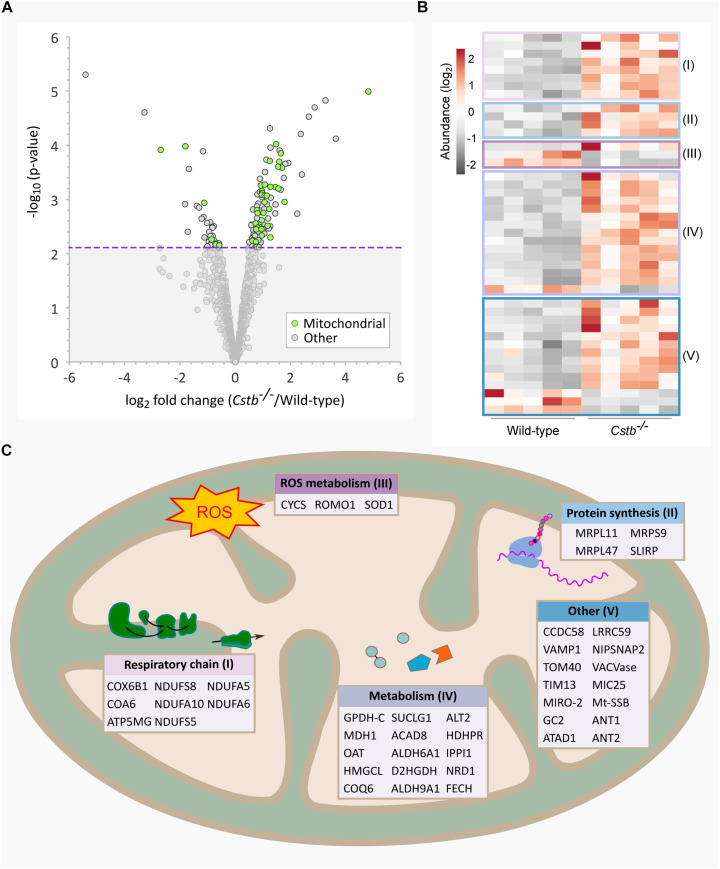
Differentially abundant mitochondrial proteins in *Cstb*^–/–^ synaptosomes. **(A)** Mitochondrial proteins (green) are plotted as −log_10_-transformed *p*-value vs. log_2_-transformed fold change (*Cstb*^–/–^/wild-type) protein abundance. The 128 DAPs significantly differing in abundance (*q*-value ≤ 0.05) are plotted above the threshold limit of −log_10_ (*p*-value) 2.1 (purple dotted line). The remaining identified proteins (1973) with a non-significant *q*-value are plotted below the threshold limit. **(B)** Heatmap of 44 differentially abundant mitochondrial proteins with 36 and 6 proteins having increased and decreased fold changes, respectively. Proteins are grouped by key processes as outlined in C. Each column represents an independent biological sample. See Supplementary Table S3 for complete list of proteins. **(C)** Differentially abundant mitochondrial proteins detected for key processes compartmentalized within the organelle.

The proteomics data was normalized, followed by missing value imputation of proteins with null-intensity in either sample group. Sample relations were examined by Spearman’s correlation analysis, hierarchical clustering, and principal component analysis (PCA) using R language and environment for statistical computing [version 3.2.2 (2015-08-14)] (R Core Team, 2016), and the Bioconductor module (version 3.2) (Gentleman et al., 2004). Statistical testing of abundance between sample groups (*Cstb*^–/–^ vs. wt) was performed using the R package ROTS (Reproducibility-Optimized Test Statistic) (Elo et al., 2008). An abundance change with a false discover rate (FDR) -corrected *p*-value (*q*-value) ≤ 0.05 was considered as significant change. Downstream analyses of the results were conducted to proteins with a *q*-value ≤ 0.05 using the following softwares and statistical tests: PANTHER Overrepresentation Test (Released 20200407) using the Gene Ontology (GO) database (Mi et al., 2013) (Released 2020-02-21), using Fisher’s exact test followed by Benjamini-Hochberg correction (FDR) for multiple testing, considering FDR < 0.05 statistically significant; the web-tool ClustVis (Metsalu and Vilo, 2015), performed using singular value decomposition; the STRING database (Szklarczyk et al., 2019), using high confidence interaction score (>0.700) as cutoff, and experiments, databases, and co-expression as active interaction sources. The softwares Cytoscape (Shannon et al., 2003) and Inkscape^2^ were used for visualization of the results. Function and localization of mitochondrial proteins for Figure 1C were manually curated.

### Electrophysiological Recordings

#### Cerebellar Slice Preparation

Mice (P14, *n* = 5 (*Cstb*^–/–^) and 10 (wt); P30, *n* = 5 (*Cstb*^–/–^) and 7 (wt); males and females) were deeply anesthetized with halothane, followed by cervical dislocation. Brains were quickly dissected and placed into ice-cold (+4°C) cutting solution with (in mM): 87 NaCl, 2.5 KCl, 0.5 CaCl_2_, 25 NaHCO_3_, 1.25 NaH_2_PO_4_, 7 MgCl_2_, 50 sucrose, and 25 D-glucose. Sagittal cerebellar slices (225 μm) were cut using a vibrating microtome (7000 SMZ-2; Campden Instruments). Slices were incubated for 1 h at +34°C in standard solution containing (in mM): 124 NaCl, 3 KCl, 2 CaCl_2_, 25 NaHCO_3_, 1.1 NaH_2_PO_4_, 2 MgSO_4_, and 10 D-glucose. The slices were maintained at room temperature until use in electrophysiological recordings. All solutions were equilibrated with carbogen (95% O_2_, 5% CO_2,_ pH 7.4).

#### Electrophysiological Recordings of Tonic GABA_A_R Currents and Effects of Blocking GAT-1

Whole-cell voltage clamp recordings were performed in a genotype-blinded manner from cerebellar granule cells, 1-2 cells per animal. Granule cells were identified based on their morphology and electrophysiological signatures (Silver et al., 1992). Currents were amplified (EPC10, HEKA Elektronik GmbH, Lambrecht, Germany) at a sampling rate of 20–50 kHz. Data were collected using Patchmaster software (HEKA Elektronik GmbH). Recordings were corrected for a liquid junction potential of 3.9 mV. Patch pipettes from borosilicate glass had open-tip resistances of 4–7 MΩ, and they were filled with a solution containing (in mM): 140 CsCl, 10 HEPES, 5 EGTA, 4 NaCl, 1 CaCl_2_, 2 Mg-ATP, 5 QX-314 bromide, pH 7.3 at +20°C with NaOH; osmolarity 284 mOsm. Recordings were done in a submerged recording chamber at +32 ± 0.5°C, and slices were constantly perfused with standard solution (3.5 ml/min). GABAergic transmission was pharmacologically isolated by blocking ionotropic AMPA and NMDA receptors using CNQX (10 μM) and D-AP5 (20 μM), respectively. GABA_A_R antagonist picrotoxin (100 μM) was used at the end of all experiments to fully block all GABA_A_R -mediated currents, including the extrasynaptic tonic current. Picrotoxin and the GAT-1 inhibitor NNC-711 (10 μM) were directly applied to the perfusate.

Cells were voltage clamped at a holding potential of −70 mV. At the beginning of each experiment, current amplitudes were measured for 3 min to obtain a stable baseline.

#### Analysis of Data

Analysis of the electrophysiological recordings was done using the WinEDR software (Dr. J. Dempster, University of Strathclyde, Glasgow, United Kingdom). The holding currents were analyzed by taking all-point histograms derived from 30-second-windows of the recording periods (baseline; 60 s after NNC-711 application; 100 s after picrotoxin application). A Gaussian curve was fitted on the all-point histograms, and the peak value was used as the holding current value for analysis (Sipilä et al., 2005). The effect of GAT-1 activity and the total tonic GABA_A_R -mediated currents were defined as the net change in holding current following application of NNC-711 and picrotoxin, respectively. The change in tonic current in the presence of NNC-711 was read from the baseline of the recording during time intervals in which sIPSCs were absent. This eliminates any influence of synaptic currents on the quantification of the peak change in tonic current (see Figure 4A). In order to normalize the amount of the extrasynaptic tonic current with regard to cell size the data is presented as pA/pF.

Statistical analysis was done using GraphPad Prism version 8 for Windows (GraphPad Software, La Jolla, CA, United States)^3^. Testing for statistical outliers was done using the ROUT method (*Q* = 1%), and significance was evaluated using the two-tailed unpaired t-test and the non-parametric Mann-Whitney *U* test. Statistical significance was defined as *p* < 0.05.

## Results

### *Cstb*^–/–^ Synaptosomes Are Quantitatively and Qualitatively Distinct

To investigate the basis for altered GABAergic signaling in *Cstb*^–/–^ mice, we analyzed the proteome of cerebellar synaptosomes from presymptomatic P14 *Cstb*^–/–^ and wt mice by LC-ESI-MS/MS. Altogether, a total of 2101 proteins were identified and quantified (Supplementary Table S1), with Spearman’s rank order correlation coefficients varying between 0.973 and 0.986 for *Cstb*^–/–^ and 0.986 and 0.991 for wt samples, indicating strong positive relationships between the biological replicates (*n* = 5 / genotype) within sample groups. Downstream analyses were carried out for proteins with a *q*-value ≤ 0.05, narrowing the sample size to 128 differentially abundant proteins (DAPs) (Supplementary Table S2). Among these was CSTB, identified solely in synaptosomes of wt preparations, thus providing additional evidence of its location and function in the synapse.

Principal component analysis (PCA, Supplementary Figure S2A) revealed the ten replicates falling into two clusters and accounting for 75.5% (PC1) and 7.1% (PC2) of the variance, thus corresponding to the variation between genotypes. Individuals within sample groups did not cluster according to sex (females and males, *n* = 2 and 3 for *Cstb*^–/–^, and 3 and 2 for wt, respectively). Protein abundance ratios were consistent across biological replicates (Supplementary Figure S2B).

### *Cstb*^–/–^ Synaptosomes Are Distinct in Mitochondrial, Ribosomal and Intracellular Transport Proteins

To predict the biological relationships between the 128 DAPs in *Cstb*^–/–^ synaptosomes, we performed Gene Ontology (GO) classification and PANTHER enrichment analyses assessing the current biological knowledge of these proteins (Supplementary Table S3).

**FIGURE 2:**
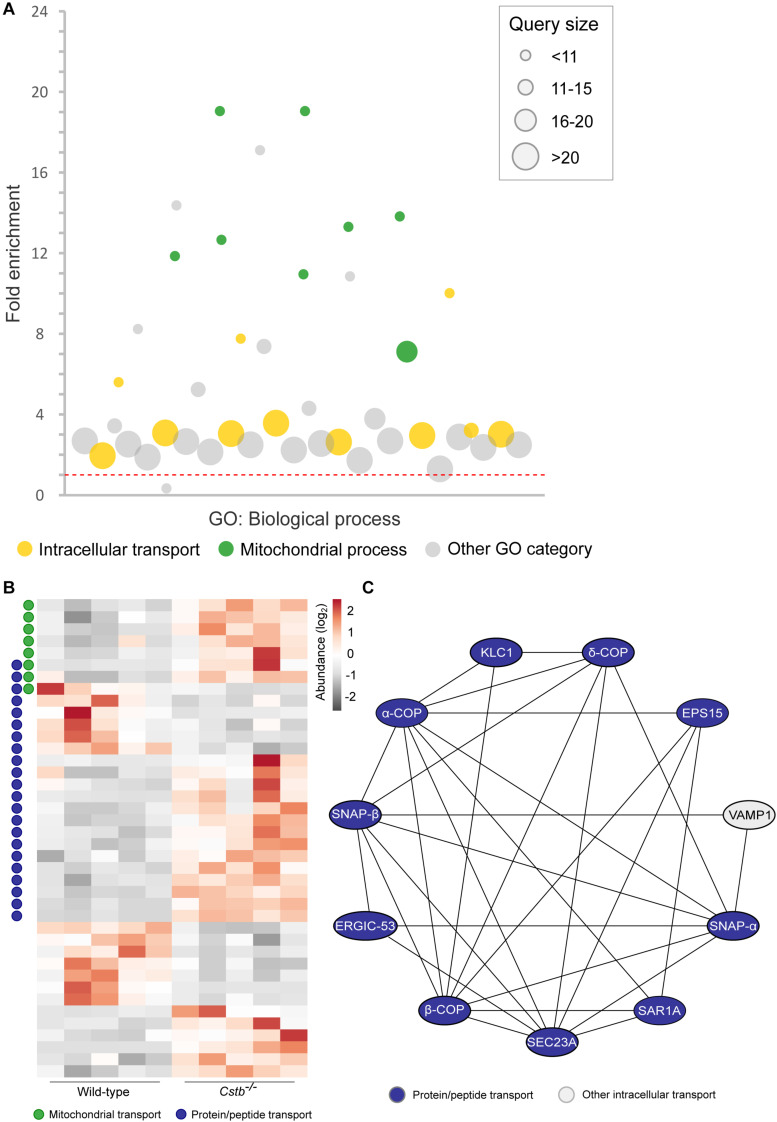
Differentially abundant intracellular transport proteins in *Cstb*^–/–^ synaptosomes. **(A)** Manhattan plot of enriched (FDR < 0.05) GO terms show a high number of intracellular transport (yellow circles; GO:0006810; GO:0071702; GO:0071705; GO:0046907; GO:0015031; GO:0015833; GO:0042886; GO:0006886; GO:0006839; GO:0048193; GO:0006888) and mitochondrial process (green circles; GO:0007005; GO:0007006; GO:0022904; GO:0022900; GO:0032981; GO:0010257; GO:0006119; GO:0033108) -related biological processes in the DAPs dataset. Each circle represents a GO term, circle size corresponding to the annotated number of proteins in the DAPs dataset (query size). GO term fold enrichment is plotted on the *y*-axis, and the threshold limit of 1 (no fold change) is plotted as a red dotted line. **(B)** Heatmap of 40 differentially abundant transport proteins in *Cstb*^–/–^ synaptosomes, with 13 and 27 proteins having decreased and increased fold changes, respectively. Proteins involved in mitochondrial or protein/peptide transport are marked with green or blue circles, respectively. Each column represents an independent sample, and each row represents a protein. See Supplementary Table S3 for complete list of proteins. **(C)** Predicted protein-protein interaction network, with a majority of interactors grouping to the GO term protein/peptide transport (blue). Networks with less than three members, or interactions below the combined interaction score threshold of 0.7, are not shown.

When examining the predicted cellular components of the DAPs, we observed a striking number of proteins that localize to mitochondria (*n* = 44) (Figure 1A). Most of these proteins were increased in abundance (*n* = 36) (Figure 1B) and the fold changes were among the highest in the dataset. The differentially abundant mitochondrial proteins affect various sub-compartments of the organelle (Figure 1C), with the highest function-related fold enrichments associated to the respiratory chain (Figure 2A and Supplementary Table S3).

Next, we investigated the predicted biological processes of the DAPs. In this category, we observed an enrichment of proteins associated with intracellular transport of organelles, proteins and other cellular substances (*n* = 40), which were grouped to several overlapping transport-related GO terms (Figure 2A and Supplementary Table S3). The majority (*n* = 27) of these proteins were increased in abundance in *Cstb*^–/–^ synaptosomes (Figure 2B), and most group to the GO terms protein and peptide, and mitochondrial transport. We also investigated the predicted relations between the intracellular transport proteins by protein-protein interaction network –analysis using the software STRING (Supplementary Table S4). Most of these interactions belong to the same network, consisting of the GO terms protein and peptide transport (Figure 2C).

**FIGURE 3:**
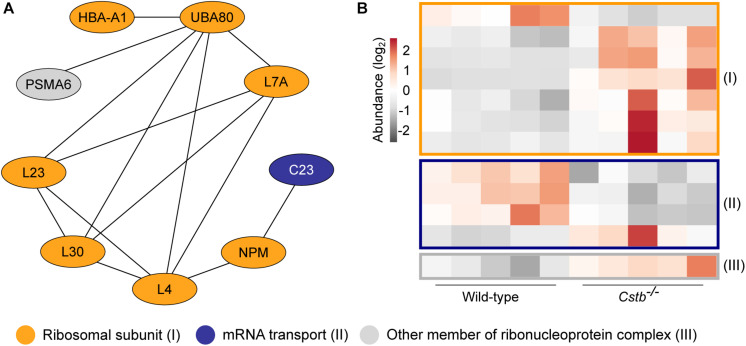
Differentially abundant proteins of the ribonucleoprotein complex in *Cstb*^–/–^ synaptosomes. **(A)** Protein-protein interaction network shows the predicted relation between ribosomal subunits (orange) and proteins involved in mRNA transport (blue). Other proteins of the ribonucleoprotein complex are plotted in gray. Protein classification was performed based on GO terms. **(B)** Heatmap of 12 differentially abundant ribonucleoprotein complex members in *Cstb*^–/–^ synaptosomes, with 4 and 8 proteins having decreased and increased fold changes, respectively. Proteins are grouped by key function. Each column represents an independent sample, and each row represents a protein. See Supplementary Table S3 for complete list of proteins.

Since axonal transport of mRNA transcripts is partly mediated by the same transport mechanisms as organelles (Kennedy and Ehlers, 2006), we asked whether the abundance of proteins involved in synaptic protein synthesis was altered. A substantial number (*n* = 16) of the DAPs were related to mRNA translation or to the ribonucleoprotein complex, as predicted by GO terms for molecular function and cellular component (Supplementary Table S3). Of these, 12 proteins were cytoplasmic and four mitochondrial, however, these two processes are functionally distinct. Nine of the cytosolic proteins were part of the same interaction network (Figure 3A and Supplementary Table S4) with the majority (*n* = 7) being structural components of the ribosome and increased in abundance in *Cstb*^–/–^ synaptosomes (Figure 3B).

### Several Differentially Abundant Synaptic Proteins in *Cstb*^–/–^ Synaptosomes

A synapse-specific location of the DAPs could provide an explanation for the previously reported alterations in GABAergic signaling. To investigate this hypothesis, we compared the DAPs dataset to the SynSysNet –online database for synaptic proteins (von Eichborn et al., 2013), and identified 31 of 128 proteins with reported synaptic location (Supplementary Table S5). Ten of these were associated with GABA- or glutamate-mediated neurotransmission (Table 1), and we studied these further by identifying their disease associations and mouse model phenotypes. GAT-1 was the only protein in the dataset exclusively associated with inhibitory GABAergic signaling, and its abundance was decreased by 0.9 fold (log_2_) in *Cstb*^–/–^ synaptosomes. Defects in GAT-1 function have previously been associated with myoclonus, epilepsy (Johannesen et al., 2018) and altered GABAergic signaling (Jensen et al., 2003), unlike any of the other synaptic proteins we identified in our analyses.

**TABLE 1:** Differentially abundant GABA and glutamate -associated proteins in *Cstb*^–/–^ synaptosomes.

Gene	Protein	log_2_ FC	Neurotransmitter	Disease association	Mouse model phenotype
*Slc6a1*	Sodium- and chloride-dependent GABA transporter 1 (GAT-1)	−0.87	GABA	MAE; ID (Johannesen et al., 2018)	Altered GABAergic signaling (Jensen et al., 2003)
*Bcan*	Brevican core protein	2.29	GABA, Glu	No reported disease association	Impaired LTP (Brakebusch et al., 2002)
*Tnr*	Tenascin-R	1.31	Glu	No reported disease association	Increased neuronal excitability (Brenneke et al., 2004)
*Napa*	Alpha-soluble NSF attachment protein	1.31	Glu	TLE (Xi et al., 2015); polymorphisms associated with PD severity (Agliardi et al., 2019)	
*Napb*	Beta-soluble NSF attachment protein	0.94	Glu	Possible contribution to EIEE1 (Conroy et al., 2016)	Epileptic seizures, ataxia (Burgalossi et al., 2010)
*Dbn1*	Drebrin	−0.68	Glu	Decreased spine plasticity in AD (Harigaya et al., 1996)	
*Actn1*	Alpha-actinin-1	−0.78	Glu	Trombocytopenia (Westbury et al., 2017)	
*Vamp1*	Vesicle-associated membrane protein 1	−1.13	Glu	SPAX1 (Bourassa et al., 2012); CMS25 (Shen et al., 2017)	Neurological defect, prewean death (Nystuen et al., 2007)
*Pura*	Transcriptional activator protein Pur-alpha	−1.35	Glu	5q31.3 microdeletion syndrome (Lalani et al., 2014)	Tremors, seizures, death at young age (Khalili et al., 2003)
*Dynll2*	Dynein light chain 2, cytoplasmic	−1.79	Glu	No reported disease association	

*GABA, gamma-aminobutyric acid; Glu, glutamate; MAE, myoclonic atonic epilepsy; ID, intellectual disability; LTP, long-term potentiation; TLE, temporal lobe epilepsy; PD, Parkinson’s disease; EIEE1, early infantile epileptic encephalopathy 1; AD, Alzheimer’s disease; SPAX1, spastic ataxia 1; CMS25, congenital myasthenic syndrome-25.*

### Unaltered GAT-1 Activity in *Cstb*^–/–^ Cerebellar Granule Neurons

To investigate GAT-1 activity in cerebellar granule neurons we turned to electrophysiology. *Cstb*^–/–^ mice are born healthy but develop progressive clinical symptoms at one month of age. Thus, we decided to test GAT-1 activity at two time points in development: P14 (presymptomatic) and P30 (early symptomatic). Tonic GABA_A_R -mediated currents are known to increase in response to GAT-1 inhibition and with increased extracellular GABA (Wall and Usowicz, 1997; Rossi et al., 2003). We inhibited GAT-1 with the antagonist NNC-711 (Suzdak et al., 1992) in voltage-clamp experiments, using the drug-induced change in tonic GABA_A_R -mediated current as a read-out of GAT-1 function.

Whole-cell voltage clamp recordings were obtained from a total of 39 cerebellar granule neurons (*n* = 6 and 13, and 8 and 12 for P14 and P30 *Cstb*^–/–^ and wt, respectively) exhibiting similar morphological and electrophysiological features. The median membrane capacitance (pF), reflecting cell size, was 3.75 and 3.70 at P14 and 3.65 and 3.90 at P30 for *Cstb*^–/–^ and wt, respectively (Supplementary Table S6). This is consistent with previous reports from cerebellar granule neurons (Kaneda et al., 1995; Brickley et al., 1996; Carta et al., 2004).

**FIGURE 4:**
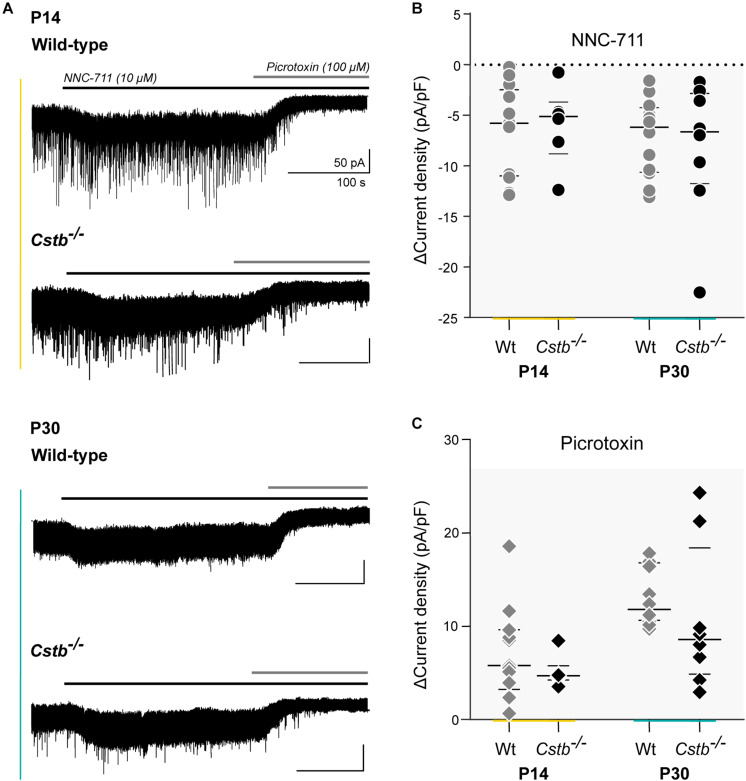
GAT-1 activity and total tonic GABA_A_R –mediated currents in cerebellar granule cells of *Cstb*^–/–^ and wild-type mice. **(A)** Experimental traces of whole-cell voltage clamped cerebellar granule neurons of P14 and P30 *Cstb*^–/–^ and wt mice. NNC-711 (10 μM) blocks GAT-1 activity. Picrotoxin (100 μM) blocks GABA_A_R -mediated tonic conductance. Recordings were obtained in the presence of CNQX (10 μM) and D-AP5 (20 μM). **(B,C)** Summary of NNC-711 and picrotoxin application. Horizontal bars represent median and interquartile range values of the change (Δ) in current density (pA/pF), induced by NNC-711 and picrotoxin application. See Supplementary Table S6 for more details.

GAT-1 inhibition caused an increase in tonic GABA_A_R -mediated currents in both genotypes (Figure 4A). However, the maximum effect of blocking GAT-1 with 10 μM NNC-711 showed large variability in both age groups (Figure 4B). A high cell-to-cell variation was also observed in the total amplitude of the tonic current as seen following its complete block by 100 μM picrotoxin (Figure 4C). The median effect of blocking GAT-1 was not different between *Cstb*^–/–^ and wt mice at either age point (Figure 4B and Supplementary Table S6). To normalize the quantifications of the GAT-1 mediated currents for each individual neuron, the ratio between GAT-1 induced maximum current (Figure 4B) and total available tonic current given by the picrotoxin-induced shift (Figure 4C) was calculated. The median values for these ratios did not indicate for differences between the two genotypes at either age point (Supplementary Table S6).

## Discussion

We undertook a proteomics approach to gain insight into the molecular basis behind altered synaptic functions in the cerebella of presymptomatic *Cstb*^–/–^ mice. Our analysis reveals that mitochondrial dysfunction might be a critical factor in the early pathogenesis of CSTB deficiency, establishing for the first time an early role for mitochondria in the molecular pathogenesis of EPM1. However, in contrast to our initial hypothesis [see (Joensuu et al., 2014], we did not detect significant changes related to GABAergic signaling.

We found one third of the differentially abundant proteins identified in the cerebellar synaptosomes of presymptomatic *Cstb*^–/–^ mice were part of the mitochondrial proteome. The fold changes of these proteins were among the highest in the dataset. Disruption of the mitochondrial proteome could affect ATP production and reactive oxygen species (ROS) scavenging, among other functions, shifting redox-balance to generate progressive mitochondrial dysfunction. In other neurological disorders, such as Alzheimer’s and Parkinson’s disease, early changes in synaptic mitochondrial density, energy metabolism, dynamics, and function have been reported to precede the onset of neurological symptoms and brain pathology (Arnold et al., 2011; Van Laar et al., 2018; Tang et al., 2019).

Considering the central role that mitochondria play in oxidative stress, it was perhaps not surprising to identify disruptions in the mitochondrial proteome because redox homeostasis was previously implicated in the disease mechanisms of CSTB deficiency (Lehtinen et al., 2009). Loss of CSTB function appears to sensitize cerebellar granule neurons to oxidative stress induced cell death with increased lipid peroxidation and depletion of antioxidants in the cerebellum of *Cstb*^–/–^ mice. Further, loss of mitochondrial membrane integrity has been reported in lipopolysaccharide stimulated primary bone marrow -derived macrophages from *Cstb*^–/–^ mice (Maher et al., 2014). A consequence of mitochondrial dysfunction is the production of ROS, which affects intracellular signal transduction pathways and generates oxidative damage (Fatokun et al., 2008). Indeed, our proteomic data show changes to the abundance of ROS-associated proteins. Notably, SOD1, an enzyme converting ROS to hydrogen peroxide limiting its potential toxicity (Wang et al., 2018) was reduced, in line with the decreased activity of SOD in cerebella of *Cstb*^–/–^ mice (Lehtinen et al., 2009). We also observed alterations to the nuclear-encoded mitochondrial protein ROMO1, and the hemoglobin subunits HBA and HBB. The latter are linked to superoxide production, increased oxidative stress, and neural pathogenesis (Nishi et al., 2008; Biagioli et al., 2009; Norton et al., 2014; Lee et al., 2018). Mitochondrial ROS has been reported to modulate GABAergic signaling in cell-type-specific mechanisms (Beltrán González et al., 2019). In cerebellar granule cells, GABA_A_R subunit α6 -mediated inhibitory currents are strengthened in response to mitochondrial ROS, although the mechanism is currently unknown (Accardi et al., 2015). Previously, we reported increased expression of both *Gabra6* and *Gabrd*, encoding the α6 and δ subunits of the GABA_A_R, in the cerebella of *Cstb*^–/–^ mice (Joensuu et al., 2014).

Our proteomic data also revealed altered abundance of key transport proteins, suggesting the potential for altered transport from the soma to the synapse. These may reflect dysfunctions in global transport mechanisms or in targeted trafficking of a subset of cargo, collectively affecting synaptic plasticity. Such mechanisms have previously been described for several neurodegenerative disorders and mitochondrial dysfunction (Liu et al., 2012). Intracellular trafficking is tied to local protein synthesis and is critical for synaptic plasticity, enabling spatio-temporal regulation of translation (Steward and Schuman, 2001; Liu et al., 2012). Consistent with this interpretation, our data revealed differential abundance of several ribonucleoproteins and ribosomal subunits in the synaptosomes of *Cstb*^–/–^ mice. Future studies will clarify the importance of these functions to the molecular pathogenesis of EPM1.

How does mitochondrial dysfunction and intracellular transport link to CSTB deficiency? Since 99% of the mitochondrial proteome is encoded in the nucleus, transport of the organelle back to the soma is required to maintain its function. This is paramount when the proteome has a differential turnover rate even among subunits of the same oxidative phosphorylation complex (Karunadharma et al., 2015). Thus, mitochondrial dysfunction could arise as a secondary effect of the cellular pathology at synapses.

The CSTB protein was robustly detected in our synaptosome preparations from wt mice, and has also been observed in synaptosomes from rat cortex and human cerebral organoids (Penna et al., 2019). The specific role of CSTB in synapses is not clear, but its function appears to be protective and crucial for synaptic physiology, as it is locally translated in synaptosomes of rat cerebral cortex (Penna et al., 2019). Furthermore, loss of CSTB function triggers a cascade of early pathological events, including mitochondrial dysfunction, compromised axonal transport, and altered local translation, leading to progressive neurodegeneration.

We did not identify factors in any major pathway that are known to specifically affect the GABAergic system. Our data, however, revealed a decreased abundance of GAT-1 in the synaptosomes of *Cstb*^–/–^ mice. GAT-1 is a crucial member of the GABA recycling system, clearing GABA from the synaptic cleft of mature GABAergic neurons, and terminating its inhibitory actions by preventing excessive activation of extrasynaptic GABA_A_Rs that mediate tonic inhibition (Guastella et al., 1990). Moreover, mutations in *SLC6A1*, the gene encoding GAT-1, have previously been linked with neurological human phenotypes, including myoclonus and epilepsy (Johannesen et al., 2018). *Gat-1* deficiency in mice increased the tonic postsynaptic GABA_A_R -mediated conductance in hippocampus (Jensen et al., 2003). No difference in GAT-1 activity in cerebellar granule cells was detected between *Cstb*^–/–^ and wt mice, which may reflect heterogeneity in the cells at P14, as the cerebellum is still developing and maturing (Altman, 1972). Indeed, GAT-1 expression in the cerebellum is dependent on the maturation of neurons. Localization of GAT-1 to the GABAergic axons does not begin until the second and third postnatal weeks, paralleling synapse formation and following the expression of vesicular GABA transporter (VGAT) (Takayama and Inoue, 2005). However, our electrophysiological analysis of GAT-1 activity at P30, when the cerebellum is further matured and *Cstb*^–/–^ mice enter the early symptomatic stage, also failed to detect any differences between the genotypes. These results are consistent with our previous studies, which showed less VGAT immunopositive puncta in the cerebellar molecular layer of P14 *Cstb*^–/–^ mice, reaching control levels by P20 (Joensuu et al., 2014). This may indicate delayed maturation of GABAergic synapses in *Cstb*^–/–^ mice. Our proteomics and electrophysiology data demonstrate that despite decreased abundance of total GAT-1 in the synaptosomes, its activity as GABA transporter in the synapse is sufficient to retain the electrophysiological properties of the cell. Its impact on other functions in the developing synapse, however, cannot be distinguished from these data, and is to be investigated in future studies.

In conclusion, our study confirms the role for CSTB in synaptic physiology and reveals a role for mitochondrial dysfunction in the early molecular pathogenesis of CSTB deficiency. The proposed hypothesis on early mitochondrial dysfunction could be the mechanism linking ROS signaling and GABAergic inhibition (Joensuu et al., 2014). Detailed understanding on how CSTB deficiency leads to mitochondrial dysfunction and the mechanisms underlying synaptic dysfunction need to be explored in future detailed studies.

## Data Availability Statement

All datasets presented in this study are included in the article/supplementary material. The mass spectrometry proteomics data have been deposited to the PRIDE database with the dataset identifier PXD019370.

## Ethics Statement

The animal study was reviewed and approved by the Animal Ethics Committee of the State Provincial Office of Southern Finland.

## Author Contributions

KG, AS, KK, BB, and A-EL contributed the study design. KG and AS performed the experiments. KG, AS, TN, and BB analyzed the data. KG, BB, and A-EL wrote the manuscript. All authors discussed and commented on the manuscript.

## Conflict of Interest

The authors declare that the research was conducted in the absence of any commercial or financial relationships that could be construed as a potential conflict of interest.

## Footnotes

1 http://proteomecentral.proteomexchange.org

2 https://inkscape.org

3 www.graphpad.com
