# Supplementary material for: Quantitative Changes in the Mitochondrial Proteome of Cerebellar Synaptosomes From Preclinical Cystatin B-Deficient Mice
Source: Front Mol Neurosci. 2020 Nov 13;13:570640. doi: 10.3389/fnmol.2020.570640 (PMC7691638; doi:10.3389/fnmol.2020.570640)
Supplement: Supplementary file 1 [file Data_Sheet_1.PDF]

Supplementary Material

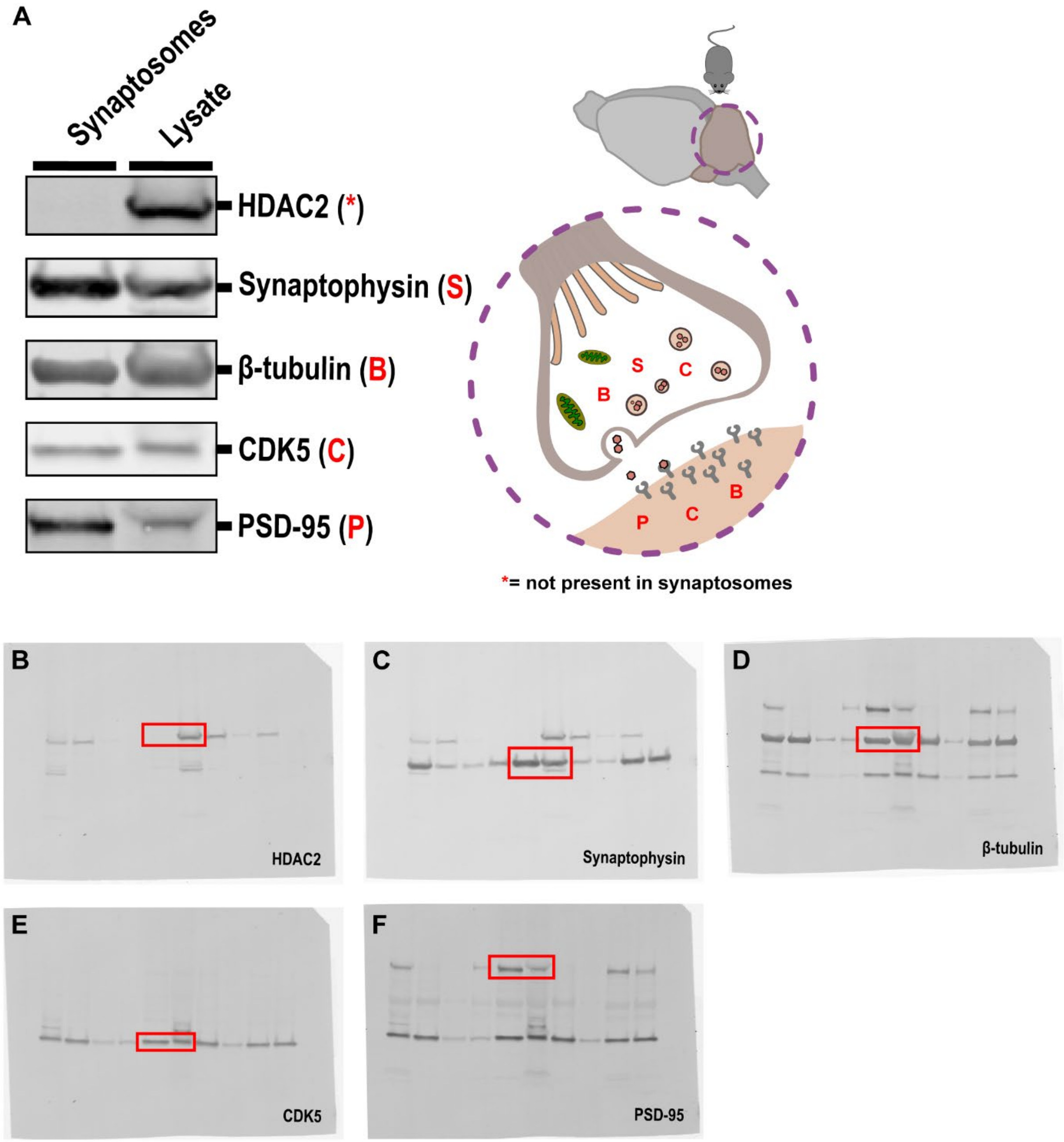

Supplemental figure 1. Representative Western blots and schematic illustration of mouse cerebellar synaptosomes. (A) Enrichment of the pre- and postsynaptic proteins synaptophysin and post-synaptic density 95 (PSD-95), and absence of histone deacetylase 2 (HDAC2) shows sample purity and synaptosome enrichment in comparison to cerebellar lysate. Equal protein loading is shown by detection of β-tubulin and cyclin-dependent kinase 5 (CDK5). **(B-F)** Full images of the Western blots used in figure A.

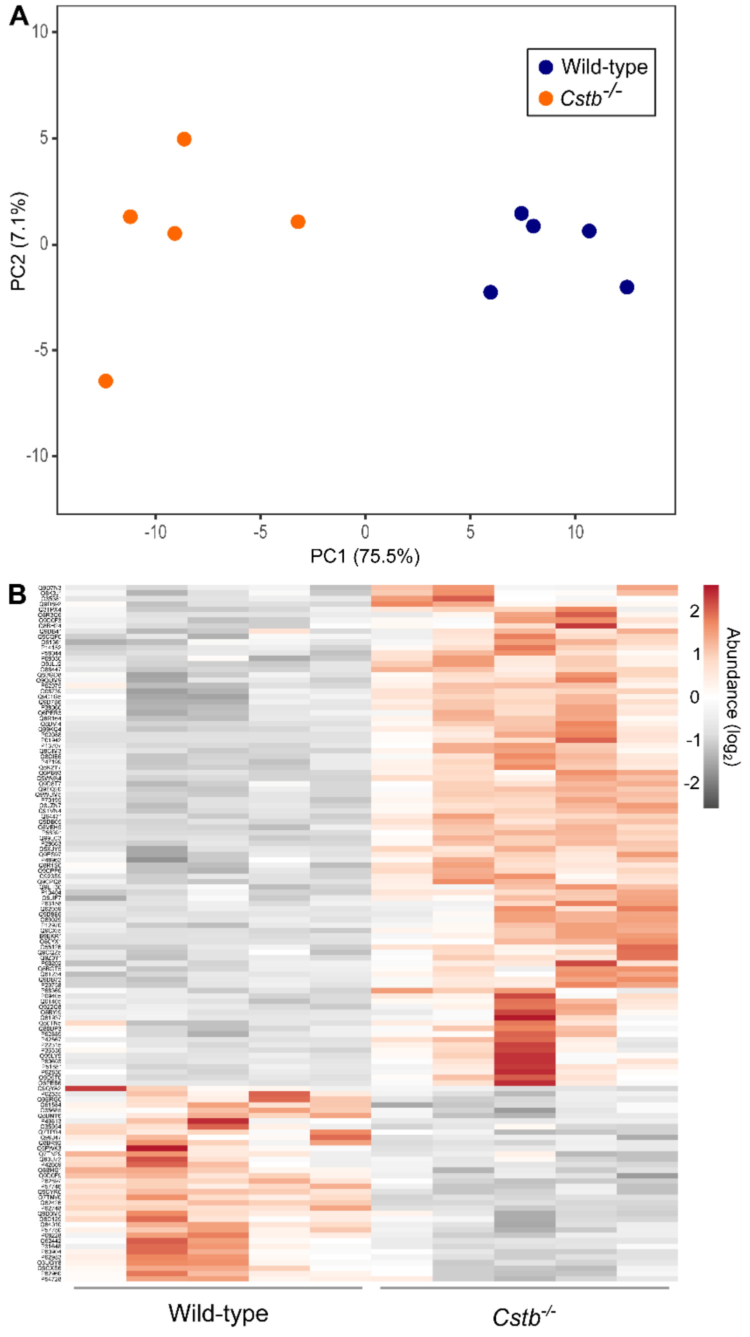

Supplemental figure 2. Overview of the 128 differentially abundant proteins (q-value ≤0.05). (A) Principal component analysis (PCA) shows the genotypes segregate into two distinct clusters. **(B)** Heatmap of the 128 differentially abundant proteins, with 92 and 36 proteins having increased and decreased fold changes, respectively. Each column represents an independent sample, and each row represents a protein. Column order is determined according to hierarchical clustering. See Supplementary table 2 for complete list of proteins.

Materials and methods

Western blot analysis

Protein samples were mixed with Laemmli buffer containing β-mercaptoethanol and boiled for 5 min at 95°C. Proteins were separated in 10% pre-casted Tris-Glycine gels (Mini-PROTEAN TGX, BioRad, CA, USA) and electroblotted to PVDF membranes (Trans-blot Turbo Transfer pack, BioRad). Membranes were blocked in 5% BSA/PBST for 60 min at r/t and incubated with primary antibodies, diluted in 5% BSA/PBST, against Synaptophysin (M0776, Dako; 1:500), HDAC2 (05-814, Millipore; 1:500), PSD95 (610495, BD Transduction Laboratories; 1:500), CDK5 (05-364, Upstate; 1:2000), and β-tubulin (T4026, Sigma Aldrich; 1:5000) o/n at +8°C. Secondary antibodies (anti-rabbit-IRDye 800CW and anti-mouse-IRDye 800CW; 926-32211 and 926-32210, LI-COR Biosciences, NE, USA), diluted 1:10 000 in 1% milk/PBST + 0.01% SDS, were incubated for 60 min at r/t. The Odyssey Infrared Imaging system (LI-COR Biosciences) was used for antibody detection.
